# Clinical Outcomes of Drug-Eluting Bead Transarterial Chemoembolization Loaded with Raltitrexed for the Treatment of Unresectable or Recurrent Hepatocellular Carcinoma

**DOI:** 10.1155/2022/2602121

**Published:** 2022-08-23

**Authors:** Yonghua Bi, Dechao Jiao, Jianzhuang Ren, Xinwei Han

**Affiliations:** Department of Interventional Radiology, The First Affiliated Hospital of Zhengzhou University, Zhengzhou, China

## Abstract

**Objectives:**

Although raltitrexed shows therapeutic effects in many types of malignant tumors, the therapeutic effects and safety of drug-eluting bead transarterial chemoembolization (DEB-TACE) loaded with raltitrexed for the treatment of hepatocellular carcinoma (HCC) are rare. This study aimed to investigate the safety and efficacy of DEB-TACE with raltitrexed-loaded CalliSpheres beads (CB) in patients with unresectable or recurrent HCC.

**Methods:**

Between May 2018 and October 2021, 41 patients with unresectable or recurrent HCC treated by DEB-TACE loaded with raltitrexed were retrospectively enrolled. The primary end points were overall survival and progression-free survival. The response evaluation criteria in solid tumors (RECIST) criteria and modified RECIST criteria (mRECIST) were used to assess the tumor response after the DEB-TACE procedure.

**Results:**

A total of 79 DEB-TACE procedures were successfully performed, and the technical success rate was 100%. The overall response rate and disease control rate assessed by mRECIST criteria were 76.9% and 88.5%, 62.5% and 70.8%, and 35.3% and 47.1%, respectively, at 1, 3, and 6 months postprocedure. The mean progression-free survival and overall survival were 21.6 ± 3.6 and 43.7 ± 5.8 months, respectively. The 6-, 24-, and 36-month overall survival rates were 86.8%, 62.7%, and 57.1%, respectively. Minor complications were observed in 21 patients (51.2%), with no treatment-related mortality or severe adverse events. The most common treatment-related complications were abdominal pain (48.8%) and nausea (29.3%).

**Conclusion:**

DEB-TACE with raltitrexed-loaded CB suggests a feasible, safe, and efficacious palliative regimen in unresectable or recurrent HCC patients.

## 1. Introduction

Hepatocellular carcinoma (HCC) is a common leading cause of cancer-related mortality worldwide [1,2]. Only about 20% of patients are diagnosed with early-stage HCC and can be treated with curative treatments, while most patients are diagnosed with late-stage HCC and its prognosis remains poor. Transarterial chemoembolization (TACE), an efficacious palliative treatment, has been performed clinically to improve survival for patients with unresectable HCC [3,4]. Rougier et al. performed TACE with raltitrexed-mixed lipiodol followed by an intraarterial infusion with oxaliplatin [5].

Nowadays, there is still no standard regimen of chemotherapy for the TACE procedure. Doxorubicin, fluorouracil, and oxaliplatin were the most common drugs used during TACE. A number of adverse effects are still observed in these drugs; thus, alternative agents are needed [6]. Raltitrexed is a quinazoline antifolate thymidylate synthase inhibitor [7] and shows therapeutic effects and safety in a number of malignant tumors, such as breast and colorectal cancers [8–10], as well as HCC [11]. Currently, drug-eluting bead TACE (DEB-TACE) has been used for many kinds of malignant tumors [12–14]. However, the clinical outcomes of raltitrexed-based DEB-TACE for the treatment of HCC are rare. Yang et al. [15] investigated the efficacy of DEB-TACE loaded with epirubicin and raltitrexed, rather than as a single agent, making it difficult to distinguish its efficacy from other drugs. This study was conducted to investigate the safety and efficacy of DEB-TACE with raltitrexed-loaded CalliSpheres beads (CB) in patients with unresectable or recurrent HCC.

## 2. Methods

### 2.1. Patients

**Figure 1:**
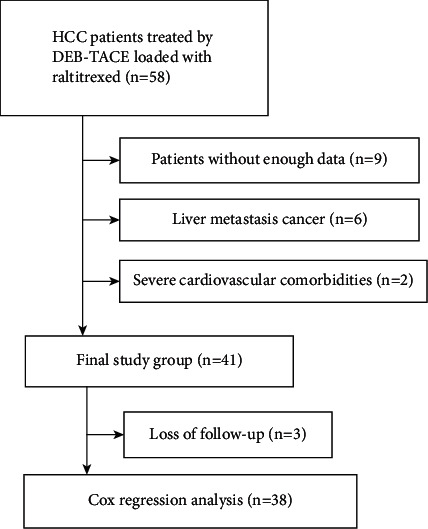
The flowchart of study population.

This was a retrospective cohort study conducted in a single center and was approved by the institutional review board of the university. Written informed consent for the DEB-TACE protocol was obtained from all enrolled patients. Patients were recruited from our department between May 2018 and October 2021 and were diagnosed with primary HCC, including both unresectable and recurrent HCC (Figure 1). Contrast-enhanced computed tomography (CT) and/or magnetic resonance imaging (MRI) were performed before DEB-TACE and during the follow-up period. For research analysis, the following preoperative clinical data were collected from the medical record systems: sex, age, Child–Pugh class, alanine aminotransferase (ALT), aspartate aminotransferase (AST), alpha-fetoprotein (AFP), platelets (PLT), tumor size, tumor number, extrahepatic metastasis, and prothrombin time (PT).

Inclusion criteria included age 18–80 years; a life expectancy >3 months; Barcelona Clinic Liver Cancer (BCLC) stages A, B, or C; and Child–Pugh class A or B. Exclusion criteria included allergy to study drugs; liver metastasis cancer; severe cardiovascular comorbidities; and coagulopathy or bleeding diathesis. The DEB-TACE treatment for HCC patients is discussed and individually tailored according to the BCLC staging system [16]. The final choice of the DEB-TACE strategy needs to consider factors such as the treatment willingness and economic ability of the patients and their families.

### 2.2. DEB-TACE Procedures

**Figure 2:**
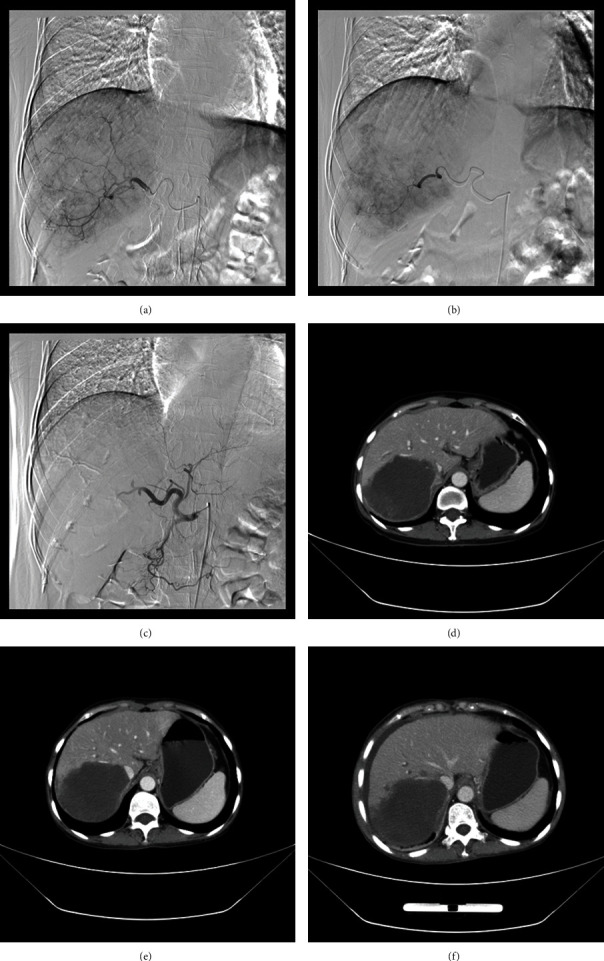
A 56-year-old female treated by CB for advanced primary HCC in the right lobe. (a) A bulky tumor staining shown in the right lobe. (b)-(c) The tumor-feeding artery was super-selectively catheterized and embolized by 300–500 *μ*m raltitrexed-loaded beads. (d)–(f) The stable tumor with no enhancement found to shrink at 1.6-, 3.6-, and 5.9-month follow-up.

The right femoral artery was punctured after local anesthesia, and a 5F catheter (Terumo, Japan) was introduced for catheterization and angiography of the hepatic artery to show tumor staining and its feeding vessels. A 2.7F microcatheter (Progreat, Terumo, Japan) was introduced for super selection of the tumor-feeding arteries. Raltitrexed (4 mg) was preloaded with CB (Jiangsu Hengrui Medicine Co. Ltd., Jiangsu, China) for 30 minutes and then mixed with iodixanol at a ratio of 1 : 1. Oxaliplatin (50–100 mg) or lobaplatin (20–40 mg) was infused into the tumor-feeding arteries. Only one bottle of raltitrexed-loaded CB (100–300 *μ*m or 300–500 *μ*m) was used for each DEB-TACE procedure; polyvinyl alcohol particles (Merit, American), gelatin sponge particles, or iodized oil (5–20 mL, GUERBET, France) were used if insufficient (Figures 2(a)–2(c)).

### 2.3. Efficacy and Safety Evaluation and Follow-Up

The primary end points were overall survival and progression-free survival. Tumor responses were assessed using the response evaluation criteria in solid tumors (RECIST) [17,18] and the modified RECIST (mRECIST) [19]. Patients were followed up by computed tomography (CT) or magnetic resonance imaging (MRI) scans at 1, 3, and 6 months after the procedure and every 2-3 months thereafter (Figures 2(d)–2(f)). A telephone follow-up was performed for all patients, with the last follow-up date of August 27, 2022. Safety and toxicity were the secondary end points. Adverse events were assessed according to the National Cancer Institute Common Toxicity Criteria (version 3.0) [20].

### 2.4. Statistical Analysis

A statistical analysis was performed using SPSS version 24.0 (IBM, Armonk, NY, USA). Overall survival was analyzed using the Kaplan–Meier analysis, followed by the log-rank test. The prognostic factors for overall survival and progression-free survival were determined by performing univariate and multivariate analyses. Statistically significant variables (*p* < 0.20) in the univariate analysis were entered into the multivariate Cox regression model. The outcomes were reported using hazard ratios and associated 95% confidence intervals (CI). A *p* < 0.05 was set as statistically significant.

## 3. Results

### 3.1. Patient Characteristics

In total, 41 patients were enrolled in this study, including 31 males and 10 females (mean age 58.4 ± 11.4 years, range 36–80 years). Detailed baseline demographics and disease characteristics are shown in Table 1. Extrahepatic metastases were present in 15 patients (36.6%). The portal vein or inferior vena cava invasion was found in 9 patients (22.0%). A total of 27 patients (65.9%) received synchronous treatments before or after the DEB-TACE procedure, including targeted therapy (*n* = 16), immunotherapy (*n* = 2), and both targeted and immunotherapy (*n* = 9).

### 3.2. DEB-TACE Treatments

A total of 79 DEB-TACE procedures were performed in 41 patients, and the mean was 1.9 ± 1.3 procedures. Twenty-one patients completed at least two cycles of DEB-TACE. All DEB-TACE procedures were successfully performed, with a technical success rate of 100%. Except for 27 DEB-TACE procedures with a CB of 100–300 *μ*m, the remaining 72 DEB-TACE procedures were performed by using CB of 300–500 *μ*m in diameter. All patients received a bottle of beads. Additional embolization was performed by gelatin sponge particles of 350–560 *μ*m during 11 procedures or polyvinyl alcohol particles of 350–560 *μ*m during 5 procedures or embolization microsphere of 300–500 *μ*m during 2 DEB-TACE procedures. Thirteen patients (31.7%) received other interventional treatments, of which 11 patients underwent c-TACE, 2 patients underwent ^125^I seed implantation in the portal vein, and 4 patients received thermal ablation (Table 2).

### 3.3. Tumor Response

None of the patients achieved a complete response after the DEB-TACE procedure if assessed using the RECIST criteria. Only 1, 4, and 3 patients achieved partial response at the 1-, 3-, and 6-month follow-ups. The overall response rates were 3.0%, 15.4%, and 16.7%, respectively, at 1, 3, and 6 months postprocedure (Table 3). The tumor enhancement decreased significantly after the DEB-TACE procedure if we evaluated the response using the mRECIST criteria. A higher overall response rate was observed, with 76.9%, 62.5%, and 35.3%, respectively, at 1, 3, and 6 months postprocedure. The disease control rates were similar, with 88.5%, 70.8%, and 47.1%, respectively, at 1, 3, and 6 months post procedure (Table 4).

### 3.4. Survival

**Figure 3:**
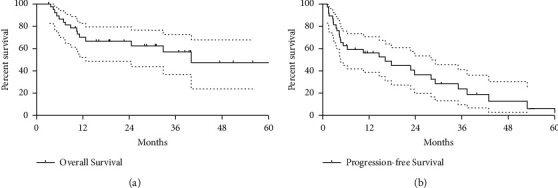
Survival curves regarding overall survival and progression-free survival. The mean progression-free survival and overall survival were 21.6 ± 3.6 months (95% CI 14.5, 28.8 months) and 43.7 ± 5.8 months (95% CI 24.2, 56.0 months), respectively.

Two patients were lost to follow-up and the remaining 38 patients were followed up, with a median follow-up length of 17.5 (9.8, 37.3) months. Sixteen patients died of tumor progression. The mean progression-free survival and overall survival were 21.6 ± 3.6 months (95% CI 14.5, 28.8 months) and 43.7 ± 5.8 months (95% CI 24.2, 56.0 months), respectively. The 6-, 24-, and 36-month overall survival rates were 86.8%, 62.7%, and 57.1%, respectively. The 6-, 24-, and 36-month progression-free survival rates were 63.0%, 37.0%, and 24.0%, respectively (Figure 3).

### 3.5. Safety and Toxicity

Minor complications occurred in 21 patients (51.2%), with no treatment-related mortality or severe adverse events. The most common treatment-related complications were abdominal pain (48.8%) and nausea (29.3%). All reported toxicities were grades 1 and 2, which were mild and manageable.

### 3.6. Prognostic Factors Analyses

**Figure 4:**
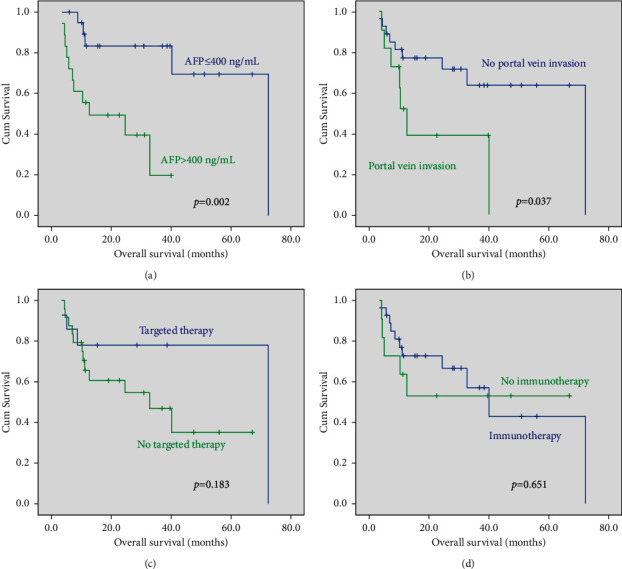
Cumulative overall survival rates and Kaplan–Meier analysis of subgroups. AFP>400 ng/mL and portal vein invasion were the independent risk factors for overall survival by the log-rank test of multivariate analysis. Targeted therapy or immunotherapy was not the independent risk factor for overall survival.

Table 4 shows the results of univariate and multivariate analyses. Moreover, Cox's proportional hazard model suggested that AFP>400 ng/mL was an independent predictive factor for progression-free survival (HR = 0.234, 95% CI: 0.063–0.869, *p*=0.030) and overall survival (HR = 0.326, 95% CI: 0.107–0.994, *p*=0.049). Targeted therapy or immunotherapy was not the independent risk factors for overall survival (Figure 4).

## 4. Discussion

The highest incidence of HCC is shown in Southeast Asia, and the major risk factor is hepatitis B virus infection [21]. Curative treatment is recommended as the first-choice treatment for patients with early HCC. In particular, liver transplant is considered in HCC patients within the Milan criteria, and radical surgical resection and ablation are curative treatments in HCC patients who are not suitable for liver transplant [22]. However, most patients are diagnosed with intermediate or advanced stage HCC and cannot be treated with curative treatments. In this case, palliative locoregional treatments (e.g., TACE and hepatic arterial infusion chemotherapy) and systemic drugs such as targeted therapy and immunotherapy can be considered. TACE is recommended as the first-line therapy for HCC patients with BCLC tumor stage B [23]. TACE is also a valid treatment option in patients with single HCC not suitable for curative treatment [22]. Oxaliplatin in combination with doxorubicin or fluorouracil is usually performed during the TACE procedure. However, these drugs are not suitable for patients with previous cardiotoxicity or high cardiologic risk factors [24], indicating a need for a new kind of agent. Raltitrexed is a thymidylate synthase inhibitor [9], which is effective in many types of tumor types [8]. Raltitrexed may theoretically serve as a substitute for fluorouracil due to the lack of cardiac toxicity [6, 24].

Nowadays, DEB-TACE has been used for many kinds of malignant tumors [25–27]. By injecting drug-eluting beads directly into the tumor-feeding artery, DEB-TACE shows significant advantages over conventional chemotherapy, which not only embolizes the tumor to promote tumor necrosis but also improves the antitumor effect and alleviates the side effects of the drugs by slowly releasing the drug. However, very few studies have reported the efficacy and safety of DEB-TACE loaded with raltitrexed for the treatment of advanced HCC. Zhao et al. [5] reported that raltitrexed plus oxaliplatin-based TACE is a safe and efficacious regimen in unresectable HCC. Yang et al. [15] reported that DEB-TACE loaded with epirubicin and raltitrexed improves the clinical outcomes and survival rate in patients with intermediate and advanced HCC. Both studies investigated the role of raltitrexed in combination with other drugs such as oxaliplatin and epirubicin, rather than as a single agent, making it difficult to distinguish its efficacy from other drugs.

In this study, the technical success rate of DEB-TACE was 100%. The mean progression-free survival and overall survival were 21.6 ± 3.6 months and 43.7 ± 5.8 months, respectively. It is reported that raltitrexed-based TACE shows a superior objective response rate than that of doxorubicin and fluorouracil-based TACE in patients with unresectable HCC. Consistent with previous studies, we also observed a higher overall response rate determined by mRECIST criteria than the RECIST criteria [5]. The complete response was almost zero if assessed by RECIST, which was similar to the previous report [11]. Besides, most patients showed a significant decrease in tumor enhancement after the DEB-TACE procedure, indicating that mRECIST may be more suitable for locoregional therapy.

Safety was also evaluated to study whether DEB-TACE loaded with raltitrexed was tolerable and safe in patients with unresectable or recurrent HCC. These data suggested that the most common treatment-related complications were abdominal pain (48.8%) and nausea (29.3%), similar to a previous study [5]. There was no treatment-related mortality or severe adverse events. All reported toxicities were grades 1 and 2, which were manageable and reversible.

Despite the advances in pharmacological and locoregional therapies, HCC is still considered to be one of the most lethal malignancies. The survival outcomes of unresectable HCC are still poor after the failure of first-line therapy due to poor knowledge of its biology and limited therapeutic options. Sorafenib is the first tyrosine kinase inhibitor used for advanced HCC patients and has been the approved treatment option for several years [28]. However, its efficacy is limited by the development of drug resistance [28]. In this study, sorafenib (*n* = 8), lenvatinib (*n* = 7), and apatinib (*n* = 10) were used. However, targeted therapy or immunotherapy was not an independent risk factor for progression-free survival or overall survival. The possible explanation is that the small sample size and the inconsistent use of targeted drugs affected the results of the statistical analysis. A combination of biologic therapy with immune checkpoint inhibitors seems to be promising for a new therapeutic strategy for patients with unresectable HCC [29]. However, the safety and efficacy are still not well established for the subset of patients, including those with preexisting inflammatory bowel disease, autoimmune disease, or nonalcoholic steatohepatitis [29].Furthermore, regorafenib, as a second-line agent, showed promising results and may represent a valuable and relatively safe therapeutic option in intermediate/advanced HCC patients after sorafenib failure [30].

This study had several shortcomings. This was a retrospective study conducted in a single center, and the sample size was too small to allow for statistically powered subgroup analysis. Multiple targeted agents and immunotherapy were used, which are likely to have an impact on efficacy outcomes and Cox regression analysis. It is necessary to study DEB-TACE in combination with different targeted agents or immunotherapy. There were selection bias and confounding factors in this study, such as combined targeted therapies, immunotherapy, ablation, and other therapies.

## 5. Conclusion

Our preliminary results suggest that DEB-TACE with raltitrexed-loaded CB is safe and tolerable in patients with unresectable HCC; further clinical studies are warranted to compare to an additional cohort receiving targeted and/or immunotherapy.

## Figures and Tables

**Table 1:** Patient characteristics at admission.

Parameters	Data
Male, *n* (%)	31 (75.6%)

Mean age, years	58.4 ± 11.4

Lesion types	
Right lobe	30 (73.2%)
Left lobe	6 (14.6%)
Bilobar	5 (12.2%)

Symptom duration, months	1.0 (0.3, 7.0)

Recurrence after surgery	5 (12.2%)

Systemic treatments	27 (65.9%)
Targeted therapy	16 (39.0%)
Immunotherapy	2 (4.9%)
Targeted and immunotherapy	9 (22.0%)

Hepatitis B/C virus infection	19 (46.3%)/3 (7.3%)

BCLC stage A/B/C	5 (12.2%)/28 (68.3%)/8 (19.5%)

Child–Pugh class A/B	30 (73.2%)/11 (26.8%)

Multinodular/bulky tumor/diffuse	9 (20.0%)/27 (65.9%)/5 (12.2%)

Portal vein or IVC invasion	9 (22.0%)

Extrahepatic sites	15 (36.6%)
Lung	2 (4.9%)
Lymph node	11 (26.8%)
Bone	1 (2.4%)
Kidney or adrenal gland	3 (7.3%)

Tumor diameter, mm	72.2 ± 34.1

a-Fetoprotein level	
Normal (<20 ng/mL)	18 (46.2%)
20–<400 ng/mL	9 (23.1%)
400–10000 ng/mL	7 (17.9%)
>10000 ng/mL	5 (12.8%)

IVC, inferior vena cava.

**Table 2:** Clinical data on DEB-TACE.

Variables	Data
Inpatient duration, days	10.0 (8.0, 14.0)
Oxaliplatin, mg	94.4 ± 15.2
Lobaplatin, mg	21.5 ± 4.3
Hospitalization cost, ×10^4^ ¥	5.9 ± 2.2
DEB-TACE procedures	1.9 ± 1.3

Additional embolization	
Gelatin sponge particles	10 (24.4%)
Embolization microspheres	2 (4.9%)
Polyvinyl alcohol particles	4 (9.8%)

Complications, *n* (%)	21 (51.2%)
Fever	4 (9.8%)
Nausea	12 (29.3%)
Vomiting	2 (4.9%)
Leukopenia	5 (12.2%)
Abdominal pain	20 (48.8%)
Raised ALT/AST	3 (7.3%)

Other interventional treatments, *n* (%)	13 (31.7%)
Conventional TACE	11 (26.8%)
^125^I seeds implantation	2 (4.9%)
Thermal ablation	4 (9.8%)

ALT, alanine aminotransferase; AST, aspartate aminotransferase; TACE, transcatheter arterial chemoembolization.

**Table 3:** Local tumor response assessed using RECIST and mRECIST criteria.

Response	RECIST criteria			mRECIST criteria		
	1 month	3 months	6 months	1 month	3 months	6 months
CR	0 (0.0%)	0 (0.0%)	0 (0.0%)	3 (11.5%)	3 (12.5%)	1 (5.9%)
PR	1 (3.0%)	4 (15.4%)	3 (16.7%)	17 (65.4%)	12 (50.0%)	5 (29.4%)
SD	26 (78.8%)	13 (50.0%)	6 (33.3%)	3 (11.5%)	2 (8.3%)	2 (11.8%)
PD	6 (18.2%)	9 (34.6%)	9 (50.0%)	3 (11.5%)	7 (29.2%)	9 (52.9%)
ORR	1 (3.0%)	4 (15.4%)	3 (16.7%)	20 (76.9%)	15 (62.5%)	6 (35.3%)
DCR	27 (81.8%)	17 (65.4%)	9 (50.0%)	23 (88.5%)	17 (70.8%)	8 (47.1%)

CR, complete response; PR, partial response; SD, stable disease; PD, progressive disease; ORR, overall response rate; DCR, disease control rate.

**Table 4:** Prognostic factors for overall survival and progression-free survival determined by univariate and multivariate analyses.

Variables	Overall survival				Progression-free survival			
	Univariate		Multivariate		Univariate		Multivariate	
	HR (95% CI)	*P* value	HR (95% CI)	*P* value	HR (95% CI)	*P* value	HR (95% CI)	*P* value
AFP ≤400/>400	**0.166 (0.046, 0.604)**	**0.006**	**0.234 (0.063, 0.869)**	**0.030**	**0.368 (0.163, 0.832)**	**0.016**	**0.326 (0.107, 0.994)**	**0.049**

Age (years) ≤60/>60	0.524 (0.178, 1.540)	0.240			1.880 (0.847, 4.171)	0.121	2.632 (0.751, 9.223)	0.130

ALT (U/L) ≤35/>35	0.648 (0.233, 1.807)	0.407			**0.373 (0.157, 0.890)**	**0.026**	0.390 (0.152, 1.002)	0.051

AST (U/L) ≤40/>40	0.658 (0.224, 1.935)	0.447			0.804 (0.362, 1.784)	0.591		

BCLC stage A + B/C	1.055 (0.294, 3.788)	0.935			0.863 (0.342, 2.174)	0.754		

Child–Pugh stage A/B	2.334 (0.520, 10.47)	0.268			1.009 (0.400, 2.542)	0.985		

DEB-TACE procedures ≤2/>2	1.098 (0.395, 3.046)	0.858			0.865 (0.405, 1.849)	0.709		

Diabetes, no/yes	0.755 (0.169, 3.372)	0.713			0.841 (0.286, 2.467)	0.752		

Diameter (cm) ≤5/>5	0.264 (0.057, 1.228)	0.090	0.561 (0.084,3.762)	0.551	**0.330 (0.119, 0.912)**	**0.033**	1.190 (0.277, 5.117)	0.816

Extrahepatic metastasis, no/yes	0.825 (0.289, 2.352)	0.825			0.477 (0.217, 1.051)	0.066	0.745 (0.253, 2.194)	0.594

Hepatitis B, no/yes	0.783 (0.275, 2.229)	0.647			0.589 (0.253, 1.374)	0.221		

Hypertension, no/yes	0.581 (0.181, 1.864)	0.362			0.623 (0.240, 1.614)	0.33		

Immunotherapy, no/yes	0.780 (0.266, 2.289)	0.651			0.810 (0.352, 1.865)	0.621		

Other treatments, no/yes	2.257 (0.615, 8.282)	0.220			2.481 (0.970, 6.343)	0.058	2.334 (0.655, 8.319)	0.191

PLT×10^9^/L	1.002 (0.997, 1.007)	0.350			**1.004 (1.000, 1.008)**	**0.033**	0.999 (0.995, 1.004)	0.796

Portal vein invasion, no/yes	**0.351 (0.126, 0.980)**	**0.046**	0.283 (0.076, 1.046)	0.058	0.561 (0.235, 1.337)	0.192	0.772 (0.201, 2.972)	0.707

PT (s) ≤14/>14	1.528 (0.430, 5.427)	0.512			2.426 (0.832, 7.071)	0.104	3.951 (0.946, 16.505)	0.060

Sex, female/male	0.177 (0.023, 1.351)	0.095	0.509 (0.059, 4.399)	0.539	0.566 (0.213, 1.504)	0.253		

Targeted therapy, no/yes	0.434 (0.122, 1.539)	0.196	0.761 (0.190, 3.048)	0.699	0.556 (0.242, 1.278)	0.167	0.860 (0.332, 2.229)	0.756

Tumor number, single/multiple	0.379 (0.126, 1.139)	0.084	0.311 (0.073, 1.327)	0.115	0.448 (0.189, 1.061)	0.068	0.252 (0.064, 1.003)	0.050

ALT, alanine aminotransferase; AST, aspartate aminotransferase; AFP, alpha-fetoprotein; PLT, platelets; BCLC, Barcelona Clinic Liver Cancer strategy; DEB-TACE, drug-eluting transarterial chemoembolization; PLT, platelet; PT, prothrombin time.

## Data Availability

The clinical data were obtained from the Interventional Department of the First Affiliated Hospital of Zhengzhou University. The data used to support the findings of this study are available from the corresponding author upon request.

## Abbreviations

TACE: Transcatheter arterial chemoembolization
DEB-TACE: Drug-eluting beads TACE
HCC: Hepatocellular carcinoma
mRECIST: Modified response evaluation criteria in solid tumors
CT: Computed tomography
MRI: Magnetic resonance imaging
BCLC: Barcelona Clinic Liver Cancer
CB: CalliSpheres beads
ALT: Alanine aminotransferase
AST: Aspartate aminotransferase
AFP: Alpha-fetoprotein
PLT: Platelets
PT: Prothrombin time.
